# Editorial: Special Issue, “Molecular Advances in Skin Diseases”

**DOI:** 10.3390/ijms232012396

**Published:** 2022-10-17

**Authors:** Naoko Kanda

**Affiliations:** Department of Dermatology, Nippon Medical School Chiba Hokusoh Hospital, Kamagari 1715, Inzai 270-1694, Chiba, Japan; n-kanda@nms.ac.jp; Tel.: +81-476-99-1111; Fax: +81-476-99-1909

The pathomechanisms of various skin diseases have recently been elucidated progressively. The therapeutic targets of certain skin diseases—inflammatory, infectious, or neoplastic—have been rapidly identified, and thus, novel target-oriented therapies have been extensively developed. In this Special Issue of *IJMS*, we have published seven research articles, four reviews, one concise communication, and two case reports regarding recent advances in the research of all fields of skin diseases from molecular viewpoints.

The gene *NIPAL4* encodes transmembrane Mg^2+^ transporter Prader–Willi/Angelman syndrome-like domain containing 4 (NIPA4), and the mutation of this gene causes autosomal recessive congenital ichthyosis characterized by aberrant barrier function and diffuse skin scaling. Marunaka et al. identified the role of Mg^2+^ homeostasis in skin barrier integrity [1]. A high concentration (5.8 mM) of MgCl_2_ induced the expression of hyaluronan synthase 2/3 and the production of hyaluronic acid in human keratinocyte-derived HaCaT cells, and also enhanced the recovery rate in the wound-healing assay. These effects of Mg^2+^ supplementation were blocked by *NIPAL4* knockdown. The results suggest that cosmetic products containing a high concentration of Mg^2+^ may be useful for enhancing moisturization and wound repair via the elevation of hyaluronic acid production in the skin.

Systemic sclerosis (SSc) is characterized by excessive collagen deposition in the skin and internal organs. Type I collagen is a heterotrimeric protein comprised of two α1(I) collagen and one α2(I) collagen polypeptides (encoded by *COL1A1 and COL1A2* genes, respectively). Sawamura et al. found that the relative expression ratio of *COL1A1* to *COL1A2* in SSc fibroblasts was significantly higher than that in control fibroblasts [2]. The local injection of *COL1A1* siRNA in a bleomycin-induced SSc mouse model attenuated skin fibrosis. The results suggest that *COL1A1* siRNA may be an efficient therapeutic strategy in SSc.

Dystrophic epidermolysis bullosa (DEB) is an inheritable blistering disease caused by mutations in *COL7A1* encoding type VII collagen. Akasaka et al. developed a novel non-invasive method for testing the consequences of mutations in DEB using *COL7A1* mRNA extracted from peripheral blood mononuclear cells [3]. This method detected aberrant transcript and splicing resulting from *COL7A1* mutation in patients with DEB. They also found that a small number of CD105^+^, CD29^+^, CD45^+^, and CD34^−^ mesenchymal stem or stromal cells expressing *COL7A1* mRNA were circulating in the peripheral blood.

Asai reviewed the structure and roles of podoplanin, a sialomucin-like type I transmembrane receptor glycoprotein [4]. In skin lesions with psoriasis, podoplanin expression is enhanced in basal keratinocytes and may contribute to epidermal hyperproliferation and the promotion of IL-17 secretion from lymphocytes. During wound healing, the podoplanin/C-type lectin-like receptor-2 interaction between keratinocytes and platelets regulates re-epithelialization at the wound edge. The podoplanin expressed by squamous cell carcinoma or melanoma promotes their migration and epithelial-mesenchymal transition, accelerating invasion and metastasis. Podoplanin expressed by peritumoral fibroblasts or keratinocytes promotes tumor progression in melanoma or extramammary Paget’s disease. Anti-podoplanin antibodies may have therapeutic potential against skin cancers.

Hoober and Eggink reviewed the structure and functions of filaggrin, a histidine-rich major protein in keratohyalin granules [5]. The human gene for its precursor, *profilaggrin*, encodes 10, 11, or 12 nearly identical repeats of filaggrin. The filaggrin is degraded to free amino acids and urocanic acids, which aid in maintaining moisture in the cornified layer. Ichthyosis vulgaris and atopic dermatitis are associated with the decrease of filaggrin. The loss-of-function mutations in the *profilaggrin* gene have been detected in the patients with these diseases and may cause the dysfunction of the surface barrier.

Vascular anomalies are classified into vascular tumors and vascular malformations, according to the International Society for the Study of Vascular Anomalies classification published in 2018. Kunimoto et al. summarized the latest findings on genetic abnormalities in vascular anomalies [6]. Causative genes for vascular anomalies are found on molecules in the RAS/MEK/ERK pathway (RASopathy) and PI3K/Akt/mTOR pathway (PIKopathy). The molecular targeting drugs for vascular anomalies, such as PI3K, BRAF, MEK, or mTOR inhibitors, have been developed, and may broaden treatment options.

Atopic dermatitis (AD) is a chronic inflammatory skin disease characterized by type 2-skewed abnormal immunity, skin barrier impairment, and intense itch. Zeze et al. showed that the expression of phosphorylated ERK was enhanced in the epidermis of skin lesions with AD [7]. Inhibitors of ERK suppressed IL-4-induced expression of CCL17/22 in bone marrow-derived dendritic cells and restored the expression of filaggrin and involucrin in keratinocytes. Topical ERK inhibitors improved AD-like dermatitis in model mice. The results suggest that ERK pathway may be involved in the pathogenesis of AD and that ERK inhibitors may be a novel therapeutic option for AD.

Tanei and Hasegawa reviewed the research progress in the pathomechanism of spongiotic dermatitis, a hallmark of AD [8]. Recent studies have revealed the importance of IgE-mediated delayed-type hypersensitivity, the Fas/Fas-ligand system, and cell-mediated cytotoxicity for inducing the apoptosis of keratinocytes in spongiotic dermatitis.

The RASopathies comprise a group of developmental disorders caused by pathogenic variants in genes encoding the RAS/MAPK pathway. Mosaic RASopathies, caused by post-zygotic variants in components of the RAS/MAPK pathway, are a molecularly heterogeneous group of neurocutaneous syndromes with high phenotypical variability. Beyens et al. presented the continuum of mosaic neurocutaneous RASopathies, showing codon 146 *KRAS* variants in individuals with oculoectodermal syndrome and isolated epidermal nevus [9]. They identified codon 146 *KRAS* variants as an additional hot spot for non-organoid keratinocytic nevi, and as a key to resolve the molecular etiology for nevus psiloliparus.

Skin aging is categorized into chronological aging and photo-aging. Xue et al. studied the anti-aging ability of chlorogenic acid, an ester of L-quinic acid and caffeic acid, on human dermal fibroblasts (HDFs) [10]. Chlorogenic acid increased Col1 expression and decreased that of matrix metalloproteinase 1/3 in UVA-irradiated HDFs by restoring TGF-β/Smad2/3 signaling, which is crucial in Col1 synthesis. Chlorogenic acid might protect skin from photoaging.

The transplantation of autologous dermal sheath cup (DSC) cells is useful for both male and female pattern hair loss. Yoshida et al. evaluated the correlations between marker gene expression in DSC cells and treatment outcomes of their transplantation [11]. Regarding the expression of smooth muscle cell markers, *MYOCD*, *CALD1* was negatively and *SRF* was inversely correlated with the treatment outcomes. The expression of dermal papilla marker *SOX2* was positively correlated with the treatment outcomes. Therapeutic efficacy of DSC cell transplantation can be predicted by evaluating their expression of these biomarkers.

Superficial epidermolytic ichthyosis (SEI) is an autosomal dominant inherited ichthyosis, caused by mutations in *KRT2*, and frequently shows erythroderma and widespread blistering at birth. Suzuki et al. reported the clinical manifestations of two patients from a Japanese family with SEI caused by a hotspot mutation, p.Glu487Lys in *KRT2*, and summarized previous reports on SEI patients with an identical mutation [12]. Among those cases, 44.4% occurred at birth, and 11.1% manifested erythroderma. Children at a high risk of SEI due to the *KRT2* mutation p.Glu487Lys should be carefully followed until 18 months of age.

Tuberous sclerosis complex (TSC) is a rare multisystemic monogenic disease caused by a germline mutation in either *TSC1* or *TSC2* genes, encoding hamartin and tuberin, respectively, followed by a second genetic/epigenetic hit inactivating the wild-type allele. Bernardelli et al. developed a mouse model for TSC by subcutaneously injecting TSC2^−/meth^ cells into nude mice [13]. The injection induced follicular neogenesis driven by mTOR hyperactivation. Further, 5-azacytidine, a chromatin remodeling agent, reverted specific pathological manifestations. Their model will shed light on new potential pharmacological approaches for TSC.

The Runt-related transcription factor (Runx) family has been suggested to play roles in stem cell regulation, tissue development, and oncogenesis in various tissues/organs. Ogawa et al. investigated the possible functions of Runx1 and Runx3 in keratinocyte differentiation [14]. Runx1 and Runx3 likely function to directly inhibit differentiation-induced expression of *keratin 1* and *keratin 10* genes, but are not involved in the regulation of keratinocyte proliferation.

In conclusion, the articles published in this issue showed us the prospects for the research and therapeutics in various skin diseases, including neoplastic, inflammatory, or genetic diseases. We appreciate the contributions of the authors to this Special Issue, and hope for the future progress of their study.
